# 

**Published:** 1894-01

**Authors:** 


MEDICAL ASSOCIATION OF GEORGIA.—The next meeting of this Association will be held in this city on April 18-19-20, with the following officers:

President, W. H. Elliott, Savannah; 1st Vice-Presihent, G. T. Miller, Americus; 2nd Vice-President, H. McHatton, Macon; Secretary, Dan H. Howell, Atlanta; Treasurer, E. C. Goodrich, Augusta. The medical profession of the State are invited to attend. The prospects are that we will have the largest meeting here in the history of the Association.

The local doctors extend a cordial invitation to all "regular" doctors of the State to visit us during the meeting.

"The Physician's Magazine," a Monthly Chronicle of the Advances of the Medical Sciences, edited by Robert C. Kenner, A. M., M, D., Louisville, Ky., and published by George F. Russell & Co., 808 First Street, Louisville, Ky., will be issued on January 1, 1894.

